# Is Neural Activity Detected by ERP-Based Brain-Computer Interfaces Task Specific?

**DOI:** 10.1371/journal.pone.0165556

**Published:** 2016-10-28

**Authors:** Markus A. Wenzel, Inês Almeida, Benjamin Blankertz

**Affiliations:** 1 Neurotechnology Group, Technische Universität Berlin, Berlin, Germany; 2 Faculdade de Ciências, Universidade de Lisboa, Lisbon, Portugal; Ghent University, BELGIUM

## Abstract

**Objective:**

Brain-computer interfaces (BCIs) that are based on event-related potentials (ERPs) can estimate to which stimulus a user pays particular attention. In typical BCIs, the user silently counts the selected stimulus (which is repeatedly presented among other stimuli) in order to focus the attention. The stimulus of interest is then inferred from the electroencephalogram (EEG). Detecting attention allocation implicitly could be also beneficial for human-computer interaction (HCI), because it would allow software to adapt to the user’s interest. However, a counting task would be inappropriate for the envisaged implicit application in HCI. Therefore, the question was addressed if the detectable neural activity is specific for silent counting, or if it can be evoked also by other tasks that direct the attention to certain stimuli.

**Approach:**

Thirteen people performed a silent counting, an arithmetic and a memory task. The tasks required the subjects to pay particular attention to target stimuli of a random color. The stimulus presentation was the same in all three tasks, which allowed a direct comparison of the experimental conditions.

**Results:**

Classifiers that were trained to detect the targets in one task, according to patterns present in the EEG signal, could detect targets in all other tasks (irrespective of some task-related differences in the EEG).

**Significance:**

The neural activity detected by the classifiers is not strictly task specific but can be generalized over tasks and is presumably a result of the attention allocation or of the augmented workload. The results may hold promise for the transfer of classification algorithms from BCI research to implicit relevance detection in HCI.

## Introduction

If a person pays special attention to a stimulus, a particular neural response is evoked that can be detected as event-related potential (ERP) in the electroencephalogram (EEG). This phenomenon is used in brain-computer interfacing (BCI) in order to establish a communication and control channel, which is purely based on neural activity and does not involve any muscle movements. Typical ERP-based BCI systems repeatedly present different stimuli one by one to a person who selects one stimulus of interest (*target*) and silently counts its appearance and ignores other stimuli (*distractors*) [1–11]. Counting helps to direct the attention to one of several stimulus types. Targets evoke a detectable neural response in comparison to distractors (an augmented late positive going centroparietal EEG component referred to as P300) [12–14].

Recently, it was suggested that BCI technology could be transferred to relevance detection in human-computer interaction (HCI), because EEG combined with an eye tracker can be used to predict which of several items displayed at the same time on the screen are task-relevant for the user [15–20]. The application of BCI technology to HCI is presumably most useful and convenient for the user if the information (e.g. about the relevance of words or pictograms) can be inferred implicitly from the neural activity when the user pursues different activities. Accordingly, the question was addressed if the detectable, target-related neural activity is specific for the silent counting task, or if it is also present in other tasks that direct the attention to target stimuli. If the activity is not task specific, it is presumably a result of the attention allocation or of the augmented workload. Generalizability of the neural patterns would be promising for the envisaged application case, where the user performs different tasks while focusing on, and expending more mental effort on relevant screen content. While silent counting is legitimate to enhance performance in most BCI applications, relevance detection is not feasible if silent counting is essential to elicit a neural response that can be detected in single (or few) trials of EEG.

## Materials and Methods

### Experimental Design

**Fig 1:**
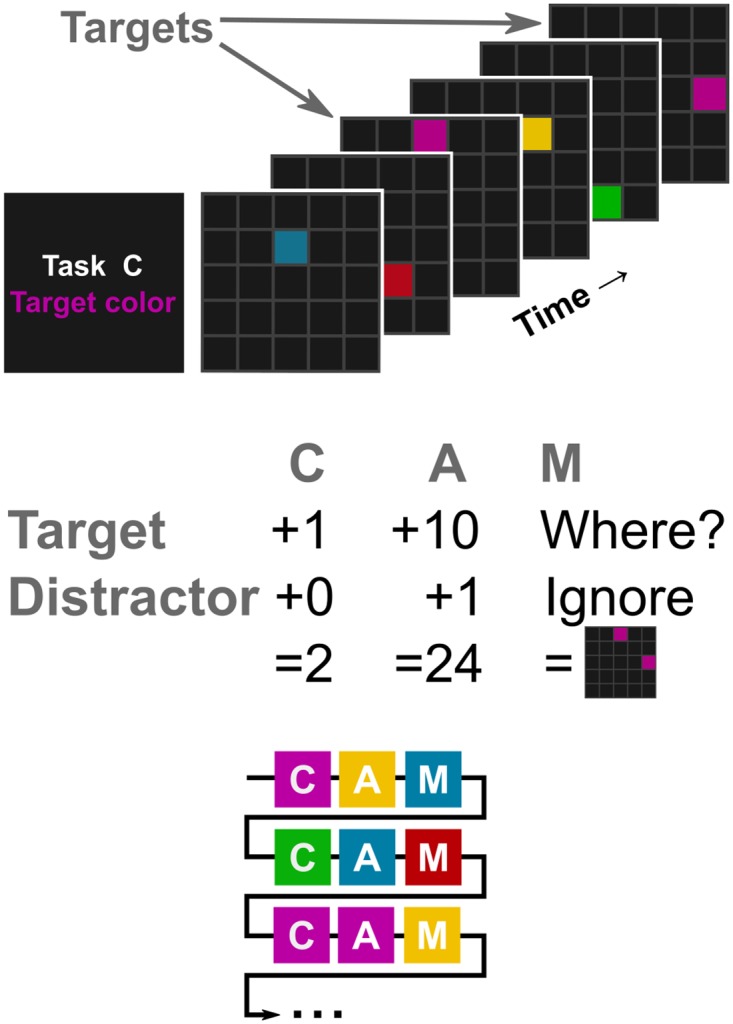
Short exemplary stimulus sequence (top), experimental tasks C, A and M (center) and sequence of the tasks and random target colors (bottom). The participants looked at a random stimulus sequence, where 47 to 50 squares of five colors flashed (with equal probabilities) in a grid for 500 ms each, interleaved by 500 ms blank screen. Before each stimulus sequence, the task and a random target color were assigned. The respective target color required a particular mental operation, depending on the task. Every participant performed task C (*counting* targets), A (*arithmetic* for targets and distractors), and M (*memorizing* target positions) twenty times each. The result had to be entered after the stimulus sequence.

Thirteen people performed a silent counting, an arithmetic and a memory task. The tasks required the subject to pay particular attention to target stimuli of a color that was randomly changed after each task repetition. The stimulus presentation was the same in all three tasks, which allowed a direct comparison of the experimental conditions. Squares in the colors magenta, yellow, red, blue and green flashed one by one for 500 ms each, interleaved by 500 ms blank screen, in a five-times-five grid in pseudo-random order and arrangement (cf. Fig 1). The probability of the appearance of each color was the same, such that the ratio of the random target color to the other colors was approximately one to four, resulting in eight to thirteen targets among the 47 to 50 colored squares in total per stimulus sequence.

Condition **C** constitutes the original version where stimuli of the target color had to be *counted* while stimuli of other colors—the distractors—could be ignored. In the *arithmetic* task of condition **A**, ‘ten’ had to be added for targets and ‘one’ for the more frequent distractors. In condition **M**, the position of the targets on the screen had to be *memorized*. The target color magenta appeared twice among four distractors in the short exemplary stimulus sequence given in Fig 1. The correct result would be 1 + 1 = 2 for condition C, 1 + 1 + 10 + 1 + 1 + 10 = 24 for condition A and ‘row 1, column 3’ and ‘row 3, column 5’ for condition M.

The task and the random target color were introduced before each stimulus sequence. After the presentation of the stimulus sequence, the result had to be entered with keyboard (numbers) or mouse (coordinates) and, finally, the correct answer was shown on the screen. The three tasks took turns and were repeated twenty times each (cf. Fig 1).

Stimuli of the target color did not stand out systematically, e.g., with respect to salience or frequency. Targets distinguished themselves only due to the preceding definition as target for the present task repetition, because each color appeared with equal probability and the target color was frequently changed.

### Experimental Setup

Participants sat at a viewing distance of approximately eighty centimeters from the screen (refresh rate 60 Hz, resolution 1920 x 1200 pixels, size 52 cm x 32.5 cm, visual angle 33° in horizontal and 22° in vertical direction) and had access to a keyboard and a mouse. EEG signals were recorded with 64 active EEG electrodes arranged according to the international 10–20 system (*ActiCap, BrainAmp, BrainVision Recorder*, BrainProducts, Munich, Germany; sampling frequency of 1000 Hz). The ground electrode was placed on the forehead, the reference electrode on the left mastoid, one of the regular EEG electrodes on the right mastoid for later re-referencing to the linked-mastoids and another electrode below the left eye for electrooculography (EOG). Electrode impedance was set at values of 5 kΩ or less, which was possible in more than 95% of the cases. If an optimal impedance between an electrode and the scalp could not be achieved despite considerable effort, this non-optimal impedance was accepted and the experiment was started. Maximum impedance at start time was 7 kΩ at the ground electrode, 9 kΩ at the reference electrode and 26 kΩ at a scalp electrode. Stimuli were presented with in-house software written in *Processing* (version 2.2.1, https://processing.org) controlled by *Matlab* (MathWorks, Natick, USA).

### Data Acquisition

Five female and eight male subjects with normal or corrected to normal vision, no report of eye or neurological diseases and ages ranging from 18 to 65 years (mean of 31.2 years) participated in the study. The tasks were introduced and trained at the beginning of the experiment of two hours. The participants gave their informed written consent to take part in the experiment. The study was approved by the ethics committee of the Department of Psychology and Ergonomics of the Technische Universität Berlin (reference BL_02_20140520).

The EEG data were re-referenced to the linked mastoids and band-pass filtered between 0.5 Hz and 40 Hz with an infinite impulse response forward-backward filter. The continuous multi-channel data were segmented in one second long epochs aligned to the flashing of targets and distractors, starting at 100 ms before the respective stimulus onset. Baseline correction was applied using the data within the 100 ms long interval before stimulus onset.

The participants repeated each task twenty times and viewed 47 to 50 stimuli per task repetition. The first eight markers per repetition that indicated the stimulus onset had to be discarded due to a jitter, i.e. an imprecision, in the stimulus presentation. As result, there were 165 ± 5 target and 648 ± 13 distractor epochs (mean ± std) available per participant and experimental condition.

### Data Analysis

#### Single-Trial Classification

The question was addressed if the neural response to target stimuli is specific to the silent counting task or if it can be also evoked by other tasks. The problem was approached by asking the subjects to perform three tasks that required to pay attention to certain stimuli. The stimuli were classified either as targets or distractors based on the immediate neural response to them. The classifiers were trained with data recorded when the subject performed one of the three tasks and tested on separate data acquired when a different task was requested. Classifiers trained in one experimental condition should be able to detect targets in different experimental conditions if the target-related neural activity is not task specific. Training and testing was performed on all possible pair-wise combinations of the three conditions. As additional reference level, every condition was inspected separately and served both for training and testing. In this case, the classification performance was assessed by splitting the data in test and training sets in a ten-fold cross-validation [21].

Spatio-temporal features for the classifications were extracted from each EEG epoch within the interval from 100 ms to 800 ms. The EEG signal was downsampled to 20 Hz in order to improve classification performance via a reduction of the dimensionality of the features [22]. A 930 dimensional feature vector was obtained for each EEG epoch by concatenating the EEG potentials measured at all 62 channels and 15 time points within the 700 ms long epoch. Classifications were performed with regularized linear discriminant analysis where the shrinkage parameter was determined analytically [23–25]. Performance was assessed with the area under the curve (AUC) of the receiver operating characteristic [26].

Single-trial classifications were performed with all samples including trials potentially corrupted by artifacts. Accepting this challenge is expected to be useful for online operation in prospective applications. Moreover, the employed multivariate methods are able to project out artifacts of various kinds.

The previously introduced classifications were conducted separately for each participant (*within*-participants). Besides, an *across*-participants classification scheme was employed in order to investigate if a transfer of the predictor is possible between subjects, which would allow to skip a time-consuming individual calibration session (cf. section ‘Discussion/Single-Trial Classification’). For this purpose, classifiers were trained on the data of all participants but one and tested on the data of the respective withheld participant. The procedure was iterated such that the data of every participant were tested. Again, all combinations of training and testing condition were assessed. Moreover, the effect of the number of training subjects on the classification performance was determined. The data of one to twelve subjects were used to train a classifier (to discriminate between targets and distractors) that was tested on the data of each withheld participant. In this analysis, all experimental conditions were merged for the sake of conciseness and in view of the envisaged application case where the users are expected to perform various tasks. The training subjects were drawn at random if there existed several possibilities.

#### Spatio-Temporal Dynamics

Additionally, the spatio-temporal dynamics of the neural responses to the flashing of target and distractor stimuli were inspected. While the main hypothesis under investigation was tested with the classification approach detailed above, this inspection allows a better understanding of the underlying reasons for success or failure of the classifications. The measured potentials were averaged over the single EEG epochs of all participants, separately for each experimental condition, class (targets/distractors), channel and time point.

The difference between the two classes was assessed by computing the correlation between the potentials of the single EEG epochs and the class label, 1 for targets and 0 for distractors, separately for each channel and time point. The yielded correlation coefficients were squared while retaining the original sign (signed r^2^ values). Again, averages across participants were calculated. The coefficients were Fisher z-transformed before averaging to make them approximately Gaussian distributed, which was reversed after averaging to bring them back to the original unit [27]. A significance threshold was not employed in order to keep the full spatio-temporal pattern including potentially subtle differences that might be exploited by the multivariate classifier, which was introduced above.

In order to ensure a clean and undisturbed visualization of the neural responses, artifact epochs had been rejected beforehand based on a maximum-minimum criterion of 100 *μ*V for the EEG channels and of 200 *μ*V for the EOG channel, within the post-stimulus interval. Around 133 ± 30 target (mean ± std) and 489 ± 150 distractor epochs remained per participant and experimental condition.

#### Behavioral Performance

It was checked that every participant complied with the instructions and performed the tasks. For this purpose, the numbers entered and the positions clicked at were compared with the correct numbers and positions and it was statistically assessed whether the results were more accurate than it can be expected if the participants answered randomly. The distances between the correct and the entered numbers were calculated in the conditions C and A. It was assessed with Mann–Whitney *U* tests if the resulting distances were significantly smaller than random distances, which had been generated by shuffling the relations between correct and entered numbers a thousand times. In the condition M, the accuracies of selecting the correct target positions were computed. Mann–Whitney *U* tests checked if these accuracies were significantly greater than random accuracies, which had been determined by moving the targets to random positions a thousand times.

Analysis and visualization of the EEG and behavioral data were performed with Python (version 3.5.2, http://www.python.org), the MNE-Python software, pandas, scikit-learn and seaborn [28–33].

## Results

### Single-Trial Classification

**Fig 2:**
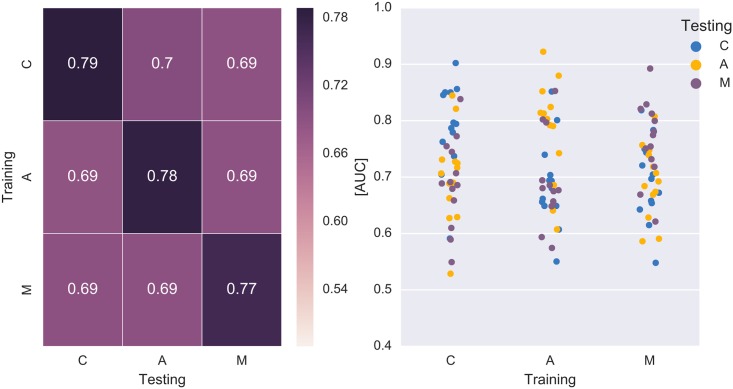
Average (left) and single participant (right) results of the classifications *within*-participants for all combinations of training and testing condition, measured as area under the curve of the receiver operating characteristic. All results were on the population level significantly better than random guessing (*p* ≤ 0.05).

Fig 2 displays the results of the within-participant classifications of target versus distractor EEG epochs. The classification performance was assessed with the AUC. This metric represents both the sensitivity and the specificity of the classifier and is insensitive to class imbalances [26]. An AUC of 0.5 constitutes the chance level of the classification. For all combinations of training and testing conditions and for every participant, the AUC was consistently better than it can be expected from random guessing. Wilcoxon signed-rank tests showed that the results were on the population level significantly above an AUC of 0.5 (*p* ≤ 0.05, Bonferroni corrected for the nine combinations of training and testing conditions).

The cross-validation results (values on the diagonal of the matrix in Fig 2) might not be directly compared with the results obtained by training on one condition and testing on a different condition (on the off-diagonal of the matrix in Fig 2).

**Fig 3:**
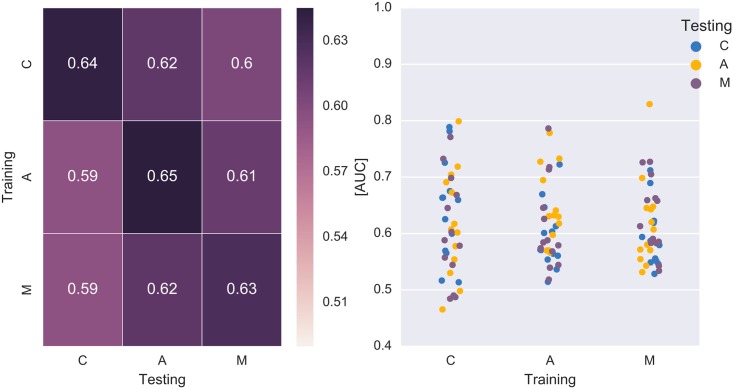
Average (left) and single participant (right) results of the classifications *across*-participants. The results were on the population level significantly better than chance, except in one case (C → M).

Fig 3 displays the results of the classifications across-participants. Classification performance was on the population level significantly better than chance in all cases but one (C → M, Wilcoxon statistic as above).

**Fig 4:**
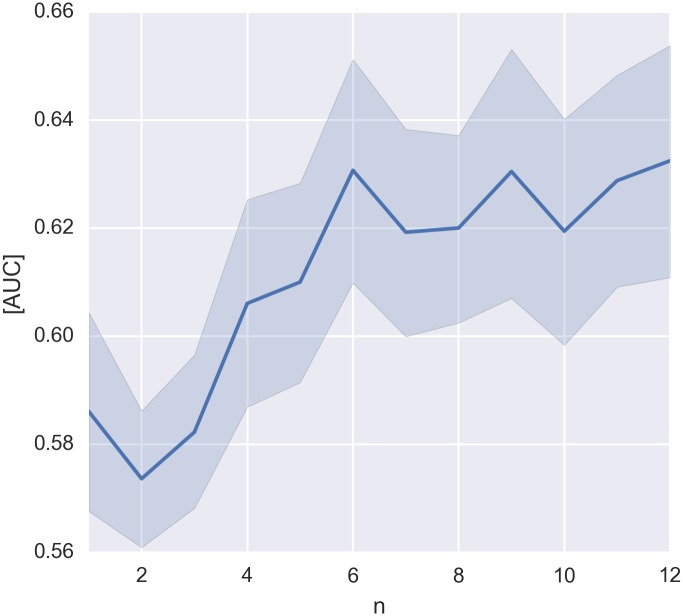
Performance (AUC) of the classification across-participants depending on the number (n) of participants used to train the classifier. The three experimental conditions were merged for this analysis (cf. section ‘Data Analysis/Single-Trial Classification’). Bootstrapping, a resampling method, was used to estimate the 68% confidence intervals (equivalent to ±1 standard deviation in the Gaussian case) of the mean across participants [34].

Data of more participants used for the classifier training resulted in a better performance when transferred to a different participant (cf. Fig 4; the three conditions were merged for this analysis as motivated in section ‘Data Analysis/Single-Trial Classification’). The number of training subjects was significantly correlated with the AUC (correlation coefficients were calculated for every subject, average: *ρ* = 0.50, t-test: *p* ≤ 0.05). Nevertheless, a ceiling effect can be observed for *n* ≥ 6.

### Spatio-Temporal Dynamics

**Fig 5:**
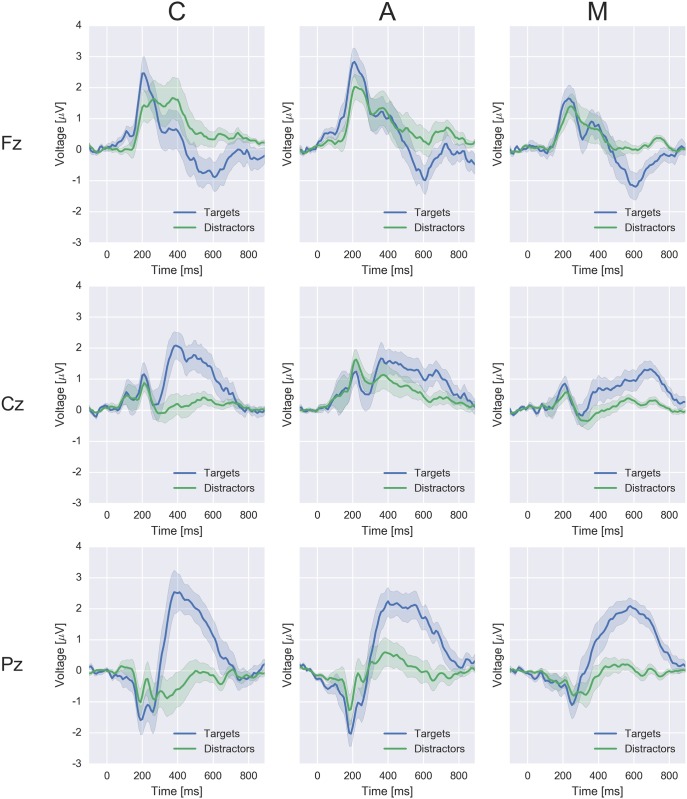
Time courses of the EEG responses to targets and distractors at the midline electrodes Fz, Cz and Pz in the experimental conditions C, A and M (averages over all epochs of all subjects). The respective stimulus-onset is situated at *t* = 0 ms. The 68% confidence intervals were calculated with bootstrapping.

**Fig 6:**
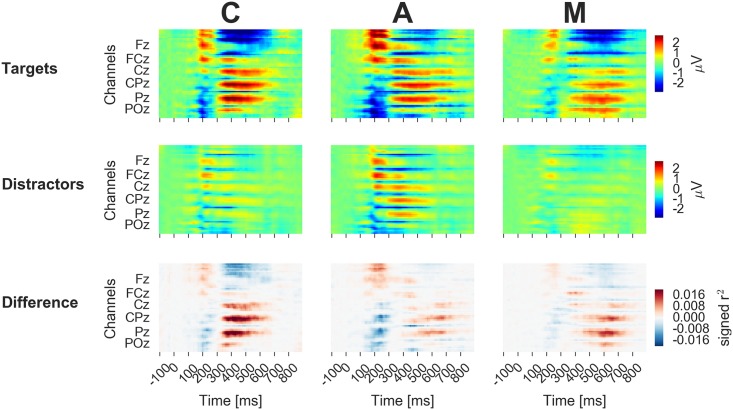
The time courses of the EEG responses to targets and distractors and of the corresponding difference (top, center, bottom) are displayed for every channel in color code.

**Fig 7:**
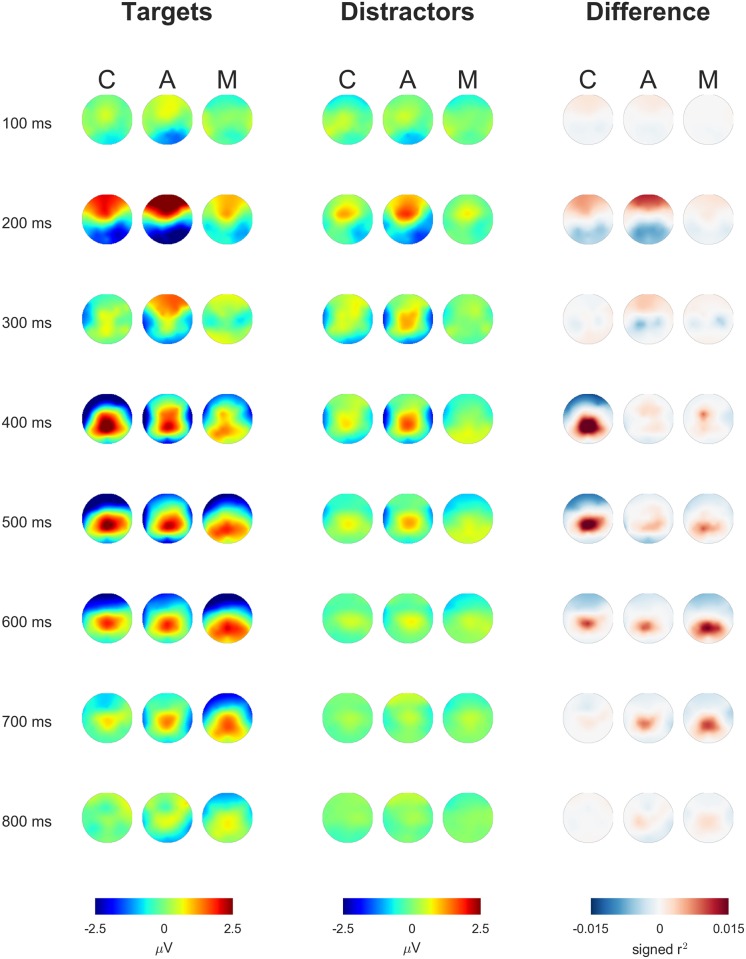
EEG responses to targets and distractors and the corresponding difference (left, center, right) are depicted as scalp topographies (head from above with the nose on top, average values over 50 ms long intervals around 100 ms, 200 ms,…, 800 ms post-stimulus-onset).

The spatio-temporal dynamics of the neural responses to the flashing of target and distractor stimuli are visualized in Figs 5, 6 and 7. Averages across participants are displayed separately for the conditions C, A and M. Fig 5 shows the time course of the EEG potential measured at frontal, central and parietal positions along the midline of the head. Fig 6 depicts the time courses at all electrodes in color code, separately for targets (top) and distractors (center). The lower row shows the difference between the two classes. Fig 7 presents the data as scalp topographies.

### Behavioral Performance

Every participant entered numbers and clicked at positions that were significantly more accurate as it can be expected by chance (*p* ≤ 0.05).

## Discussion

### Single-Trial Classification

EEG epochs that were either aligned to targets or to distractors could be discriminated significantly better than it can be expected by chance (AUC of 0.5) for all combinations of training and testing conditions (within-participant classifications; cf. Fig 2). Discrimination based on EEG data was not only possible in the classic counting variation (C) but also when both targets and distractors required arithmetic (A) or when the positions of the targets had to be memorized (M).

Each classifier could predict targets in every experimental condition and not only in the condition where it had been trained. This successful transfer suggests that a substantial part of the neural activity evoked by targets is neither specific to the silent counting, nor to the arithmetic, nor to the memory task. Both the target recognition itself, as a result of the attention allocation, and the augmented cognitive effort are equally plausible causes for the findings, because targets required a more demanding task than distractors (at least in the conditions C and M where distractors could be simply ignored).

Tailoring the classifier to each individual person, as it is typically done in BCI experiments, would be a hindering factor for the application in HCI. A time-consuming calibration session constitutes a hurdle for the users to adopt EEG-based technology for the every-day interaction with a computer. Interestingly, however, it was possible to skip the individual classifier training and predict the task-relevant stimuli with a classifier that was trained on the data of other participants (across-participants classifications; cf. Fig 3) even if the performance was significantly inferior (*p* ≤ 0.05, Wilcoxon signed-rank test) to the classification within-subjects (cf. Fig 2). Acquiring data of more participants improved the predictive performance until a ceiling level was reached for *n* ≥ 6. (cf. Fig 4). Transfer learning methods could further improve the transferability between subjects [35–40].

### Spatio-Temporal Dynamics

The patterns in the neural data that allow differentiating between targets and distractors were inspected in order to uncover the reason for the successful classifications. Targets evoked, in all experimental conditions, an augmented late positive component in comparison to distractors in particular at the midline centroparietal and parietal electrodes (cf. Figs 5, 6 and 7), which is typical for the P300 wave [13].

Some differences between the conditions can be noted (cf. Figs 5, 6 and 7): condition C, the classic variation with silent counting, featured a comparably large difference between the potentials evoked by targets and distractors. Condition A shows a comparably large late positive deflection for distractors. In this condition, all stimuli including the distractors required arithmetic and, thus, a certain amount of attention and neural processing. Finally, the discriminative neural activity lasted longer in A and M than in C (cf. Fig 7). Presumably, the memory encoding was more variable in time in these two conditions.

### Behavioral Performance

The behavioral results show that all participants complied with the instructions and performed the tasks.

### Limitations

Single stimuli popped up in succession in this experiment. However, it can be expected that several words or pictograms are shown in parallel in a more realistic setting. The combination of EEG with an eye tracker would allow to relate the neural activity to each pictogram or word [15–20]. Eye movements towards the items could be used as time points of reference for the EEG segmentation in epochs, instead of the onset of stimuli popping up on the screen. With this approach, a relevance score could be assigned to every item displayed.

We showed that the detectable neural activity evoked by targets is not specific to any of the three well-defined tasks employed in the experiment. However, it still has to be shown that relevance information can be collected implicitly in the background during the ‘natural’ interaction with a computer in the absence of precisely defined tasks.

Moreover, the stimuli used here were squares and differed only in their color. The decision if a stimulus was a target was simple and could be performed immediately. In contrast, various pictograms and words can be presented on the screen in a realistic scenario. The decision if a pictogram or a word is of interest can need sometimes less and sometimes more time. Accordingly, a variable latency of the neural response can be expected, which makes relevance estimation based on neurophysiological data more difficult [18, 19].

All stimuli were similar with respect to their salience in this experiment. Yet, in a more realistic scenario, particularly salient but not necessarily relevant stimuli could elicit a passive P300, which would result in false positive estimates (even though the passive P300 is evoked rather by auditory stimuli than by visual stimuli) [41–43].

### Possible Application in the Future

Decoding the cognitive state of computer users from neuro-, peripheral-physiological or behavioral measurements (such as EEG, electrodermal activity, facial electromyography, eye movement patterns, or pupil size) became more and more of interest recently [17–19, 44–55]. The resulting information, e.g. about the user’s attention allocation and interest, is implicitly contained in the sensor data, can be recorded in the background without any effort on the part of the user, and could augment ‘traditional’ input devices such as mouse and keyboard. Until recently, the physiological measurement devices were bulky and expensive and the set-up was inconvenient and time consuming. Yet, the situation is improving at present due to technological innovations such as miniaturized, gel-free, and in-ear as well as around-ear electrodes [56–61] and a considerable drop in the price of eye trackers [62]. Further impulses can be expected from large tech companies that launch wearable physiological sensors as parts of their products, like heart-rate sensors in smart watches, or that are working on miniaturized glucose sensors in contact lenses.

## Conclusion

Based on EEG data, screen content could be classified as task relevant or irrelevant, even when different mental operations were performed than during classifier training. The results suggest that the neural activity detected by the classifiers is not strictly task specific, and that presumably attention allocation or cognitive effort can be inferred from the EEG data, at least under the controlled conditions of this experimental study. This outcome may hold promise for a future technology transfer from brain-computer interfacing, where the users typically count the stimuli of interest, to relevance detection in human-computer interaction, where the users do not limit themselves to pursuing a specific activity.
